# Tumor Diagnosis against Other Brain Diseases Using T2 MRI Brain Images and CNN Binary Classifier and DWT

**DOI:** 10.3390/brainsci13020348

**Published:** 2023-02-17

**Authors:** Theodoros N. Papadomanolakis, Eleftheria S. Sergaki, Andreas A. Polydorou, Antonios G. Krasoudakis, Georgios N. Makris-Tsalikis, Alexios A. Polydorou, Nikolaos M. Afentakis, Sofia A. Athanasiou, Ioannis O. Vardiambasis, Michail E. Zervakis

**Affiliations:** 1 School of Electrical and Computer Engineering, Technical University of Crete, 73100 Chania, Greece; 2 Areteio Hospital, 2nd University Department of Surgery, Medical School, National and Kapodistrian University of Athens, 11528 Athens, Greece; 3 Chania General Hospital Saint George, 73100 Chania, Greece; 4 Medical School, National and Kapodistrian University of Athens, 11528 Athens, Greece; 5 Department of Electronic Engineering, Hellenic Mediterranean University, 73133 Chania, Greece

**Keywords:** brain tumor diagnosis, MRI, T2-SWI, computer aided diagnosis, DWT, CNN

## Abstract

Purpose: Brain tumors are diagnosed and classified manually and noninvasively by radiologists using Magnetic Resonance Imaging (MRI) data. The risk of misdiagnosis may exist due to human factors such as lack of time, fatigue, and relatively low experience. Deep learning methods have become increasingly important in MRI classification. To improve diagnostic accuracy, researchers emphasize the need to develop Computer-Aided Diagnosis (CAD) computational diagnostics based on artificial intelligence (AI) systems by using deep learning methods such as convolutional neural networks (CNN) and improving the performance of CNN by combining it with other data analysis tools such as wavelet transform. In this study, a novel diagnostic framework based on CNN and DWT data analysis is developed for the diagnosis of glioma tumors in the brain, among other tumors and other diseases, with T2-SWI MRI scans. It is a binary CNN classifier that treats the disease “glioma tumor” as positive and the other pathologies as negative, resulting in a very unbalanced binary problem. The study includes a comparative analysis of a CNN trained with wavelet transform data of MRIs instead of their pixel intensity values in order to demonstrate the increased performance of the CNN and DWT analysis in diagnosing brain gliomas. The results of the proposed CNN architecture are also compared with a deep CNN pre-trained on VGG16 transfer learning network and with the SVM machine learning method using DWT knowledge. Methods: To improve the accuracy of the CNN classifier, the proposed CNN model uses as knowledge the spatial and temporal features extracted by converting the original MRI images to the frequency domain by performing Discrete Wavelet Transformation (DWT), instead of the traditionally used original scans in the form of pixel intensities. Moreover, no pre-processing was applied to the original images. The images used are MRIs of type T2-SWI sequences parallel to the axial plane. Firstly, a compression step is applied for each MRI scan applying DWT up to three levels of decomposition. These data are used to train a 2D CNN in order to classify the scans as showing glioma or not. The proposed CNN model is trained on MRI slices originated from 382 various male and female adult patients, showing healthy and pathological images from a selection of diseases (showing glioma, meningioma, pituitary, necrosis, edema, non-enchasing tumor, hemorrhagic foci, edema, ischemic changes, cystic areas, etc.). The images are provided by the database of the Medical Image Computing and Computer-Assisted Intervention (MICCAI) and the Ischemic Stroke Lesion Segmentation (ISLES) challenges on Brain Tumor Segmentation (BraTS) challenges 2016 and 2017, as well as by the numerous records kept in the public general hospital of Chania, Crete, “Saint George”. Results: The proposed frameworks are experimentally evaluated by examining MRI slices originating from 190 different patients (not included in the training set), of which 56% are showing gliomas by the longest two axes less than 2 cm and 44% are showing other pathological effects or healthy cases. Results show convincing performance when using as information the spatial and temporal features extracted by the original scans. With the proposed CNN model and with data in DWT format, we achieved the following statistic percentages: accuracy 0.97, sensitivity (recall) 1, specificity 0.93, precision 0.95, FNR 0, and FPR 0.07. These numbers are higher for this data format (respectively: accuracy by 6% higher, recall by 11%, specificity by 7%, precision by 5%, FNR by 0.1%, and FPR is the same) than it would be, had we used as input data the intensity values of the MRIs (instead of the DWT analysis of the MRIs). Additionally, our study showed that when our CNN takes into account the TL of the existing network VGG, the performance values are lower, as follows: accuracy 0.87, sensitivity (recall) 0.91, specificity 0.84, precision 0.86, FNR of 0.08, and FPR 0.14. Conclusions: The experimental results show the outperformance of the CNN, which is not based on transfer learning, but is using as information the MRI brain scans decomposed into DWT information instead of the pixel intensity of the original scans. The results are promising for the proposed CNN based on DWT knowledge to serve for binary diagnosis of glioma tumors among other tumors and diseases. Moreover, the SVM learning model using DWT data analysis performs with higher accuracy and sensitivity than using pixel values.

## 1. Introduction

The most frequent forms of brain tumors are meningioma, glioma, and pituitary.

Meningiomas are the most common non-glial tumor of the central nervous system (CNS). Meningiomas are slow-growing tumors that arise from the meningothelial cells of the arachnoid. Meningiomas may be found along any of the external surfaces of the brain as well as within the ventricular system, due to arachnoid cap cells or meningocytes trapped in the cranial sutures during remolding of the brain at birth. Some meningiomas may display atypical imaging characteristics that may be diagnostically challenging. A number of benign and malignant pathologies may also mimic some of the features of meningiomas [1]. Rare cases of mixed angiomatous and microcystic meningioma are multiple tiny intralesional cysts and entrapped peritumoral cyst formation [2]. The MRI features of meningiomas are discussed in [3].

Pituitary tumors are noncancerous (benign) tumors grown in the pituitary gland. The pituitary gland is located behind the back of the nose. Superiorly, its border is the optic chiasm. Pituitary tumors do not spread to other parts of the body. The pituitary gland controls most of the body’s endocrine functions by means of the hypothalamic–pituitary axis. The pituitary tumors can cause the pituitary gland to make too few or too many hormones, causing problems in the body. In [4] is discussed the various imaging modalities for the pituitary gland.

Gliomas can be found in any region of the brain and are much harder to detect when they are lower grade. Gliomas have highly heterogeneous appearance and shape, with variable degrees of infiltration, atypia, and mitotic activity. Given the heterogeneous nature of glioma cell populations, some areas of the tumor display an infiltrative growth pattern and may not be enhanced. This tissue heterogeneity makes the current role of MRI on glioma evaluation even more challenging [5].

The diagnosis and classification of a brain tumor usually involves brain MRI scans in all three planes: axial, sagittal, and coronal, a neurological examination, and a biopsy if it can be performed safely. Magnetic resonance imaging (MRI) offers more detailed information on brain structure than computed tomography (CT) or ultrasound images. MRIs do not require ionizing radiation like CT does. The MRI generates tissue contrast based on differences in the magnetic properties of hydrogen atomic nuclei. MRI can easily differentiate between solid and cystic lesions and between different soft tissues, such as white and gray matter. Since one of the most important features of a healthy brain is its image symmetry in the axial and coronal directions, MRI is an imaging tool for diagnosing brain tumors, epilepsy, neurological diseases, etc. Asymmetry of pixel intensity along an axial MR brain image indicates a pathological brain. In the MRI image, the non-healthy area is segmented where pixel intensity is higher or lower both row-wise and column-wise in the image matrix. The differentiation in pixel intensities provides information about disease burden and lesion load.

For MRI scanning of the brain, different modalities of MRI scans respond to the different biological information in the images. The different modalities of MRI are T1, T2, T2-FLAIR, gradient imaging, diffusion weighted image (DWI), functional image, and diffusion tensor imaging. The DWI exploits the random Brownian motion of water molecules within a voxel. From DWI can be calculated the apparent diffusion coefficient (ADC), which is the measure of the magnitude of water molecules. The ADC values less than thresholds are indicating pathologies [6]. T1 MRI provides a clear view of brain anatomy and can reveal damage in brain injury when the damage is extensive. Gadolinium-based contrast agents shorten T1 relaxation time and increase tissue contrast by highlighting areas where contrast has leaked from the blood–brain barrier into interstitial tissue, resulting in parenchymal enhancement. This breakdown of the blood–brain barrier is an important feature that occurs in both tumors and non-neoplastic diseases [7]. Blood products and mineralization cannot be distinguished on T1 images, as both appear dark on magnitude images. In contrast to T1-type MRIs, T2-type allows visualization of severe diffuse axonal injury, as expected after severe traumatic brain injury. T1 images highlight fat tissue within the body and eliminates water signals. T2 images highlight fat and water within the body. Abnormally low signal relative to surrounding tissue on T1 images, or abnormal brightness on T2 images, suggests a pathological process such as cancer, trauma, or infection. Low grade gliomas usually present rather homogeneous structures with no contrast encashment or peritumoral edema, high signal intensity in their T2 type, and no contrast enhancement in their T1 type. On the other hand, high grade gliomas present heterogeneous or ring-shaped contrast or patterns in their T2 type.

The manual inspection of MR images by eye specialists is a time-consuming routine. The MRIs are diagnosed by radiologists, and the results are sent to the doctors. Since radiologists are often faced with vast amounts of MRI data showing multiple complex tumors or only few slices are affected, there is a higher risk of error diagnosis.

In the last decade, the computer aided diagnosis (CAD) tools based on AI for automated classification and grading of gliomas using MR images is one of the most challenging tasks in medical image analysis. The ultimate goal of the automated medical detection could be to assist the clinical decision-making process. Since 2018, the European Union General Data Protection Regulation has required that AI or other systems’ CAD tools should be able to explain their decisions. The future challenge will consist of finding the best combination between human and automated intelligence, taking into account the capacities and the limitations of both [8].

Since 1998, the Computer Vision, Virtual Reality and Robotics in Medicine (CVRMed), Medical Robotics and Computer Assisted Surgery (MRCAS), and Visualization in Biomedical Computing (VBC) have promoted the multidisciplinary research fields of clinicians, bioscientists, computer scientists, engineers, and physicists who are contributing to medical image computing, computer-assisted intervention, and medical robotics. Since 2004, CVRMed and VBC have merged into the MICCAI Society. Since 2012, the Multimodal BraTS challenges have been focusing on the evaluation of state-of-the-art methods for the segmentation of brain tumors in multimodal MRI scans [9,10,11,12]. BraTS’19 intended to experimentally evaluate the uncertainty in tumor segmentations. In 2021, [13,14] reported the results of the comparison between different methods that participated in 2012, 2013, 2015, 2016, and 2017 BraTS challenges. The focus of BraTS 2022 is to identify the current state-of-the-art segmentation algorithms for brain diffuse glioma patients and their sub-regions.

Researchers who are interested in review articles on brain tumor classification using CNN can study the introduction and related works in [15,16]. Moreover, in [15,16] AI-based approaches are proposed for brain tumor grading/classification. The authors in [15] are using a combination of Deep Learning (DL) and six Machine Learning (ML) training models (a total of seven AI models) and four CV protocols proving that DL methods outperform ML. The highest classification accuracy (100%) was achieved for the two-class data (normal vs. tumorous) with all cross-validation protocols. The various CAD tools that have been established to classify brain diseases using MRIs can be separated into two main groups: (i) ML models and (ii) DL models.

Different kinds of network architectures, such as convolutional neural networks (CNNs) [17,18], joint convolutional neural networks–recurrent neural networks (CNN-RNNs) [19], and generative adversarial networks (GANs) [20] have been utilized for classification systems for a multitude of computer vision tasks, i.e., cloud shape, brain tumor, and intracranial hemorrhage identification. The embedding of these models has led to significant performance gains in medical CAD.

CNN architecture including Transfer Learning (TL) of existing networks has the purpose of building up CNN with higher classification performance of CAD systems. Nevertheless, in the literature there are no in depth studies of CNN improvement in CAD applications by applying TL. In [21] the author presents a review of research focusing on different TL models classifying MR images of brain tumors, using TL of the existing networks VGGNet, Alexnet, and Resnet. The authors of [22] propose a CNN for MRI brain tumor classification and compare results with the most important literature studies using transfer learning networks. The results of [22] show that the proposed approach outperforms the deep neural networks based on existing TL networks. In general, the DL models are improved when they are based on CNNs and Discrete Wavelet Transform (DWT) [23,24,25,26]. The DL models based on CNNs with DWT kernels are a promising combination for brain MRI classification [27].

In recent paper reviews between various ML models (from PubMed, MDPI, Springer, IEEE, Science Direct, Hindawi) it has been observed that for medical diagnosis the traditional ML Support Vector Machines (SVMs) and Artificial Neural Networks (ANNs) are superseding in most of the studies in all the frameworks [26,27,28,29,30,31]. The experimental results in [31] show that traditional ML SVMs achieve higher performance on small sample data sets compared to DL framework on large sample data sets.

In general, different potential variables may be used in image classification, including spectral analysis, transformed images, textural information, etc. Designing wavelet-based CNNs helps use high-frequency components essential for classifying images. Usually, the MRI images are fed to the CAD models in the form of pixel values and in only a few studies in the form of knowledge extracted by applying DWT MRI analysis [32,33,34,35]. In [33] the DWT is combined to ANN and SVM classifiers. In [33,34] the DWT is used as input to an ANN and CNN. More often, the DWT analysis is used as input data for time-continuous signal cases in industrial diagnosis [36,37,38], for image restoration [39], image segmentation [40], and medical diagnosis [41,42]. In [43] the DWT data are input in a genetic algorithm for MRI classification. The authors of [44] studied how to embed wavelet transform into CNN architecture to reduce the resolution of feature maps while at the same time increasing the receptive field in order for object classification and image restoration. In [45], the wavelet transform is studied as an auxiliary element in deep networks. In [46] a multilevel 2-DWT-based feature matrix has been studied for classification of MR and CT images.

It is beyond this paper’s scope to provide a detailed review on comparison among ML methods. The proposal of this work is mainly the assessment of the possible higher performance of a CNN binary classifier tuned to diagnose glioma brain tumors by using DWT data analysis as a reasoning-based method to control the loss of information in the pooling layers of the CNN model. Moreover, a comparison between SVMs with and without DWT data analysis, is introduced.

The following contributions are achieved: (i) A robust CNN architecture is developed for automated binary classification of the glioma brain tumor among other tumor types and other diseases. (ii) Reduction of information loss in the pooling layers (at the expense of our CNN target) by using as input the MRIs of type T2-SWI sequences parallel to the axial plane, compressed into DWT form, thus achieving higher performance in glioma binary classification compared to a CNN that is based on pixel intensities. (iii) Lower performance of the traditional ML SVM by using DWT data instead of pixel intensities, as well as lower performance compared to a CNN.

The rest of the manuscript is organized as follows: In Section 2, materials and methods have been described. The results and the discussion of this study are in Section 3. Conclusions and future scope are mentioned in Section 4.

## 2. Materials and Methods

**Figure 1:**
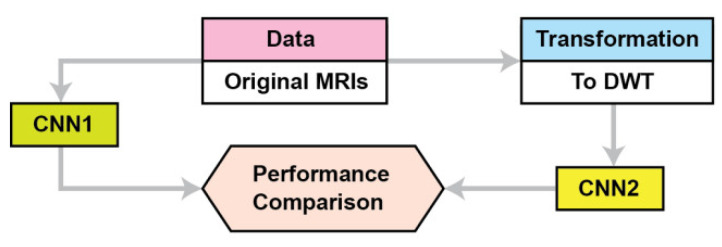
The proposed approach.

This section describes the materials and methods used in this study. Figure 1 summarizes the proposed approach. The proposed CNN is named CNN2, while CNN1 is a conventional CNN for the performance comparison. Because the application of transfer learning is based on CNNs pre-trained on images different than MRIs we propose a CNN architecture where its training is not based on transfer learning. We compare our CNN’s performance as the data used for training are DWT data instead of pixel intensities. The proposed CNN2 accepts as input the concatenate of all the frequency components’ outputs from 3 level DWTs of the original images; in contrast, the CNN accepts as input the original images (pixel intensities). Section 2.1 describes the data used, and Section 2.2 describes the proposed CNN architecture and the baseline methods for DWT of MRIs.

T2-type MR images are used instead of other types (i.e., T1) because MR signal values in T2 imaging have a larger dispersion compared to the very narrow dispersion in T1, giving us a statistical advantage in the process of the training algorithm. Moreover, T2 images enable higher resolution due to the signal creation process.

### 2.1. Data

The MRIs of type T2-SWI sequences parallel to the axial plane scans used in this study were selected manually from two datasets by our team’s expert doctors, in order to include visible pathological legions. The diagnosis of each pathological scan is independently evaluated by the experienced neurosurgeon author. All are MRIs are from different male and female adult persons, with a mean age of 34 years (range 19–65 years). One dataset originated from the Greek public St. George General Hospital, and the other from BraTS and ISLES challenges from2016 and 2017 [45]. All the MRI scans that are showing healthy cases and various diseases (other than tumorous) were generated at the Greek public St. George General Hospital, available in jpeg format, 240 × 240 isotropic pixels, where all are not skull-stripped. A skull-stripping tool, based on a combination of edge detections, morphological functions, and thresholds, is applied to these pictures. The BraTS dataset describes a collection of brain tumor MRI scans acquired from 19 different centers under different equipment and imaging protocols, all available in NIfTI format, of 512 × 512 isotropic pixels, pixel size was 0.69 mm × 0.69 mm, where all are skull-stripped. The scans used were acquired in different MR systems but all operating at a 1.5 T field strength.

The MRIs show tumors of different types, shapes, and dimensions, are heterogeneous in appearance, and show various other diseases, as shown in Table 1. A total of 572 images T2 MRI of the axial plane are collected, originating from 572 different patients. Each of the selected images shows one or more of the following—healthy tissue, hemorrhagic foci, edema, ischemic changes, non-enhancing tumor, necrosis, enhancing tumor, etc. All of our MRI scans of tumors were acquired from the multimodal segmentation challenge of BraTS.

As shown in Table 1, the testing set, named as Set2, includes original data of balanced 190 cases. For the performance evaluation, each feature scenario used the Set2 of T2 MRIs. The total scans (one of each patient) are divided into five folders, out of which the four with 382 scans are used for training and the remaining one with 190 scans is used for testing. All the details about the data used can be seen in Table 1. The MRIs used have been fully anonymized, converted in the same format and pixels’ size, and all have been skull-stripped.

A total of 39.5% of the training data are scans showing various diagnosed brain pathologies. Furthermore, 16.5% of the training data are scans showing glioma tumors, where the pathological areas of longest axis range from 1.4 mm (2 pixels) to 71.5 mm (105 pixels). Of the group, 10% of the tumor scans are selected to indicate the presence of tumors in ambiguous cases. Lastly, 44% of the training data are scans showing non-pathological scans.

Our data augmentations were applied using TorchIO [47].

#### Preprocessing

Since the scans are 2D images represented by different image matrix sizes, we adjusted the images’ matrix [48]. The images’ matrix and the annotation metadata of each scan are saved in files of DICOM format. The images are reproduced using a data augmentation technique.

### 2.2. Data Prevalence

In order to control the evaluation procedure, to ensure that precision does not depend on prevalence, the precision is normalized to a prevalence of around 50%. Prevalence is defined as in Equation (1):

(1) Prevalence=∑contition positive/∑total population

Thus, we have introduced the ratio 56% for the number of cases in the disease control group and the ratio 44% for the number of other cases in the “healthy” control group, in order to establish the Negative Predictive Value (NPV) and the Positive Predictive Value (PPV) equal to the Prevalence of the diseases in the studied datasets. Scans from the same patient are not used for both sets, and there is no overlap in content between the training and the testing sets.

### 2.3. The CNN Base Model Architecture

Many DL-based computer-aided diagnosis systems have been recently developed for the automatic diagnosis of brain tumors in MR images. Because of the rapid growth of DL algorithms in computer vision, DL methods have improved. Convolutional neural networks (CNNs) are a powerful tool within DL that can learn from experience and achieve a significant performance improvement in image classification and 2D object detection. They can classify data/images, identify image features anywhere in the image, and can provide accurate results in diagnosing medical images. Using CNN, it automatically learns the features that are important for making correct predictions on its own. A CNN trained with a large amount of data can achieve comparable accuracy to experienced physicians.

The learning processes of CNNs are still not transparent. CNNs are considered black-box models due to their nonlinear mapping and unclear working mechanisms [49]. CNNs adaptively learn spatial hierarchies of characteristics from gridded input information (i.e., signals, images). Two sets of network variables should be carefully tuned, namely, network parameters and hyperparameters. Network biases and weights are network parameters that are tuned by minimizing the error between network outcome and data labels during the training stage. Hyperparameters are the selected values of learning rate, the number of neural network hidden layers, the number of neurons, the activation functions, and the number of training epochs. They have a direct impact on the training behavior as well as the CNN model’s performance. However, there is a limitation in exploring hyperparameter space, the manual tuning of DL network’s hyperparameters is a common practice in the literature. Hyperparameter optimization has shown to be promising in improving the performance of DL networks for classifying images [50,51].

Each of the CNN’s hidden layers adaptively performs feature extraction from the input information by performing computations. These layers include the convolutional layer, pooling layers, and dense layers. Τhe role of the convolutional layer is to convolve the input information and extract feature maps. The number of convolutional layers that need to be added to a neural network to achieve the desired learning level can be determined using theoretic information quantities such as entropy, inequality, and mutual information among with the inputs to the network. The information convolving is carried out by sliding a group of small-sized filters (kernels)—each of which contains a sufficient number of adaptively learnable weights—over the input information, implementing elementwise multiplication at each possible position of the image. The kernel size of convolutional layers only affects the learning speed of the network. The number of generated feature maps is called convolutional layer depth and is defined by the number of kernels. Then, a pooling layer is used to minimize the size of the feature maps. The classification and identification of objects are done by the last dense layer. Although the pooling operation of the CNN reduces the size of the features on each layer for steps of more than one, it results in a loss of information that can affect the recognition performance. As evidenced in the literature, the CNNs fail to attain accuracy comparable to near-perfect. In [52], the wavelet pooling is studied as an alternative to traditional neighborhood pooling.

The transfer learning method is very commonly and successfully used for first-round CNN training, where the CNNs use a deep CNN model that has been previously trained as a basic framework. Pre-trained models, e.g., ResNet50 and VGG19, are pre-trained on a large dataset (e.g., ImageNet). In our case we did not use Transfer Learning because the MRI scans should have been adjusted according to the pre-trained model’s input size. Moreover, the pre-trained models have been trained on different target images, not similar to MRIs. When the initial and target problems are not similar for the first training round, then the Transfer Learning is ineffective for the first training [53].

The CNN’s input information can be any type of signal (i.e., original audio signal, DWT of an audio signal, Fourier transformation of an audio signal, pixel intensities of an image, etc.). In the literature review it is observed that untraditionally, the CNNs for image analysis are using as input the pixel intensities of the images, which are labeled at the image level. In our study, the proposed CNN extracts its knowledge from the DWT analysis of the MRIs.

#### CNN1 Model Architecture

**Figure 2:**
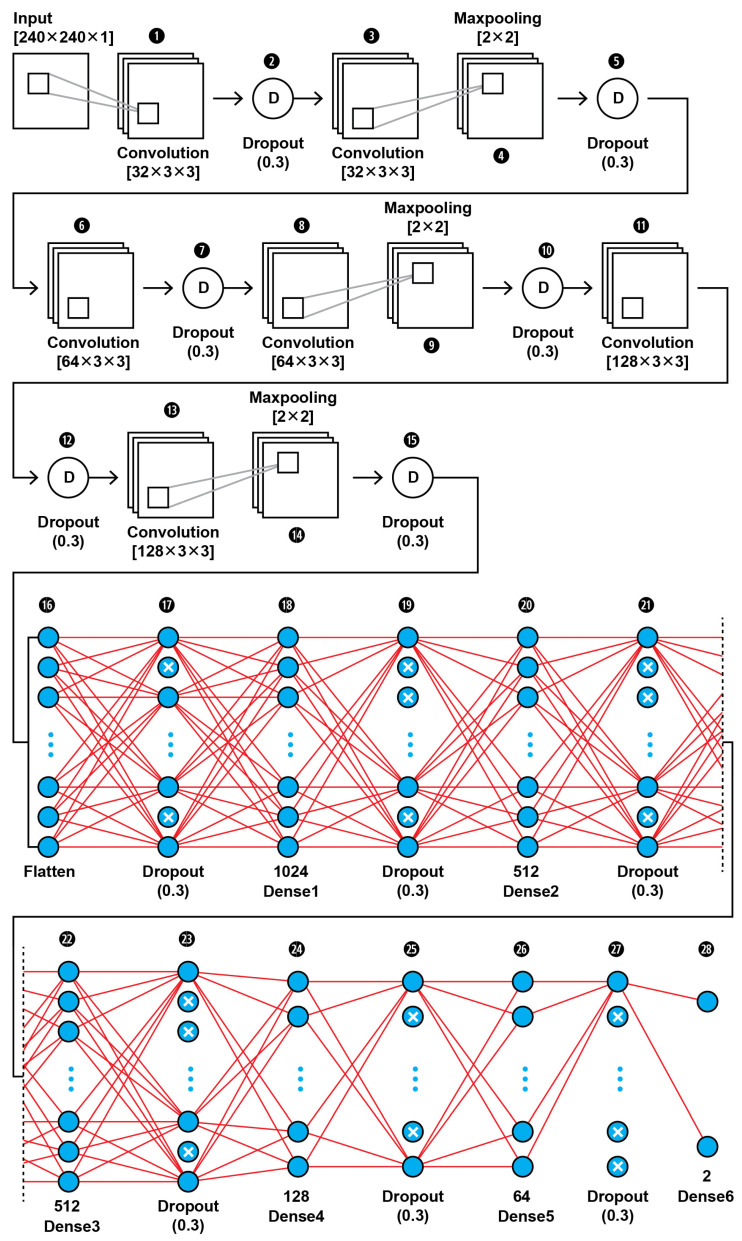
The proposed architecture of our CNN1 using as input the images’ pixel intensities.

**Figure 3:**
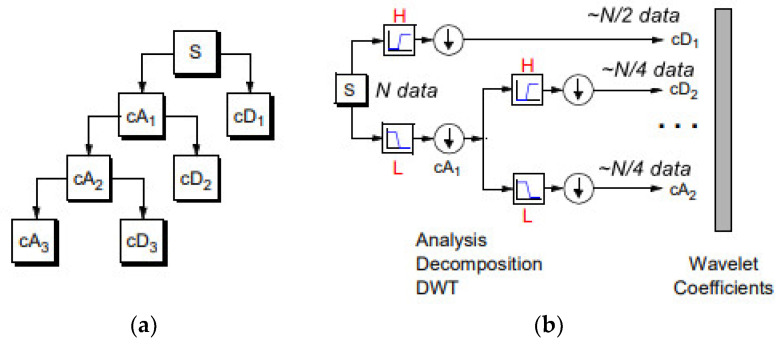
(**a**) 1D DWT decomposition level 3 of a signal s, of length N: cA1 represents the DWT approximation coefficients of level 1, cD1 represents the DWT detail coefficients of level 1. (**b**) The signal of N data, after passing the first DWT level High filter (H) is analyzed to a vector of length N + 2n − 1, and then the two approximation coefficients are down-sampled denoted by 1 (down arrow) to (N − 1)/2 + n~N/2, cD1. The next step splits the cA1 coefficients applying the same scheme to N/4 approximation coefficients, cD2 (down-sampling means throwing away every second data point). The signal can be composed by the coefficients cA2, cD1, cD2.

The topology of our proposed CNN1 (as shown in Figure 2) was found to be the best fit for this classification task through trial and error. In Figure 3, the input layer holds the augmented images as pixel intensities reshaped and normalized from 0–255 to 0–1, in the dimensions of (240,240,1). In the data extraction phase of the CNN1, a total of six convolutional layers with same kernel size of 3 × 3 and different filter number, including 32, 64, and 128, were used in this 28-layer CNN1 architecture. Dropout layers were using during both the feature extraction phase of the CNN1 and the classification phase in order to prevent the case of overfitting. The hyperparameters are set before the training operation. The hyperparameter optimization used for this training is presented in Table 2. Our CNN1 configuration is shown in Table 3.

### 2.4. DWT and CNN MRI Feature Extraction

Our enhanced CNN2 binary classifier accepts as input the spatial features obtained from two-dimensions (2D) by using the Haar wavelet decomposition features up to three levels.

A discrete wavelet transformation of any signal can be viewed as passing the original signal through a quadrature mirror filter (QMF) that consists of a pair of a low-pass filter (H) and of a high-pass filter (G). Wavelet functions are mathematical functions that decompose data into different frequency components and then study each component with a resolution matched to its scale. The wavelet function has the properties of time shift and scalability. Notations about discrete wavelet theory are referred to in [54].

The basic 2D objects are shown in Table 4. The original image is convolved along two vertical directions by low pass and high pass filters. The images obtained are down-sampled by columns indicated by 2. Down-sampled columns means that only even-indexed columns are selected. The resultant images are then convolved again with high pass and low pass filters, so now down-sampled by rows are denoted by 1; this ultimately yields four sub-band images of half the size of original image. Image analysis based on wavelets is typically implemented by memory-intensive algorithms with high execution time. In the usual DWT implementation, the image decomposition is computed by means of a convolution filtering process and so its complexity rises as the filter length increases.

In the regular DWT computation, the image is transformed at every decomposition level first row by row and then column by column.

In our study, the wavelet decomposition is done using the simplest wavelet Haar-1 up to 3 levels. The Haar wavelet transform preserves the energy of a signal. Similar to the other wavelets transforms, it decomposes the discrete signal into two subsignals of half length. One subsignal is a running average (approximation sub-band), the other subsignal is a running difference (detail sub-band).

Equation (2) calculates the case of an image being decomposed into a first level approximation component $Y_{A}^{1}$ ($Y_{A}^{1}$ contains low frequency components of the image) and detailed components $Y_{horizontal}^{1}$, $Y_{vertical}^{1}$, and $Y_{diagonal}^{1}$, corresponding to horizontal, vertical, and diagonal details (they contain high frequency components):

(2) $$Y=Y_{A}^{1}+Y_{horizontal}^{1}+Y_{vertical}^{1}+Y_{diagonal}^{1}$$

Equation (3) calculates the case when the image decomposition is repeated up to *P* levels, the export of image **Y** can be written in terms of *N*^th^ approximation components as in Equation (3):

(3) $$Y=Y_{L}^{P}+\sum_{k=1}^{P}\{Y_{horizontal}^{k}+Y_{vertical}^{k}+Y_{diagonal}^{k}\}$$

Hence the size of the approximation component obtained from the first level decomposition of an *N* × *N* image is *N*/2 × *N*/2, second level is *N*/4 × *N*/4 and so on.

**Figure 4:**
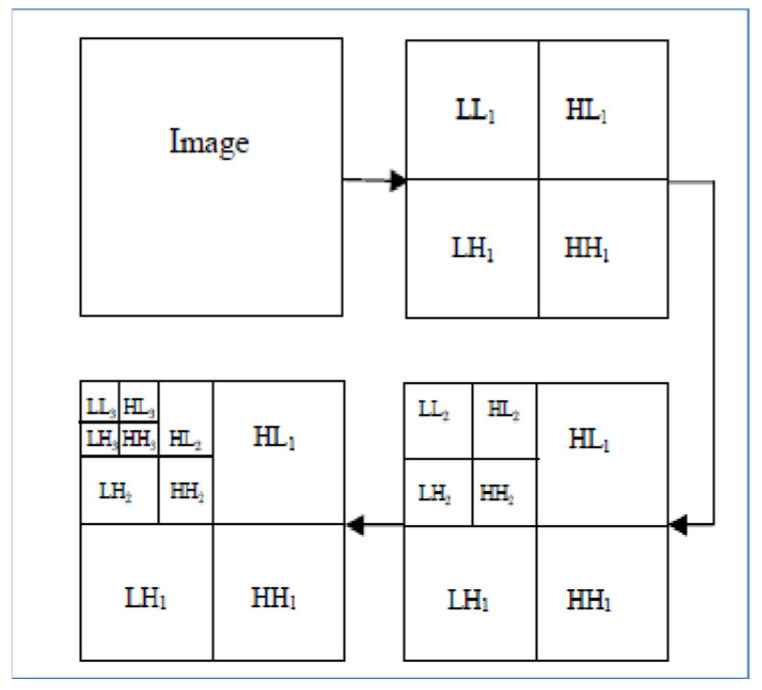
Each MR image decomposition creates 10 sub-bands in the third level. The LL (Aj) sub-band is the approximation image which is used for 2D DWT calculations in the next level.

The MR images level 3 decomposition by DWT creates 10 sub-bands, see Figure 4. Because approximation sub-bands provide more information than detailed coefficients, we use the approximation image for the next level analysis. As Figure 4 illustrates, the result is four sub-band (LL: low–low, LH: low–high, HL: high–low, HH: high–high) images obtained at each level. Among them, three sub-band images LH (Dhj), HL (Dvj), and HH (Ddj) are the detail images along horizontal, vertical, and diagonal directions, respectively.

DWT mathematically involves four fixed convolution filters with stride 2 to implement the down-sampling operator. Given an image **Y**, the (i, j)^th^ value of **Y**_LL_, **Y**_LH_, **Y**_HL_, and Y_HH_ after 2D Haar transform can be written as in Equations (4)–(7):

y_LL_(i, j) = y(2i − 1, 2j − 1) + y(2i − 1, 2j) + y(2i, 2j − 1) + y(2i, 2j) (4)

y_LH_(i, j) = −y(2i − 1, 2j − 1) − y(2i − 1, 2j) + y(2i, 2j − 1) + y(2i, 2j) (5)

y_HL_(i, j) = −y(2i − 1, 2j − 1) + y(2i − 1, 2j) − y(2i, 2j − 1) + y(2i, 2j) (6)

y_HH_(i, j) = y(2i − 1, 2j − 1) − y(2i − 1, 2j) − y(2i, 2j − 1) + y(2i, 2j) (7)

For our 2D DWT analysis, the DWT is applied to each dimension separately, i.e., the rows and columns of the image are separately undergone through the 1D DWT to build up the 2D DWT.

In the present work, we have computed the approximation coefficients of level 3 decomposition and these coefficients are fed to the CNN as the primary feature vector for each MR image. We selected as mother wavelet algorithm the simplest wavelet Haar, Since the Haar wavelet is orthogonal and symmetric in nature, it gives good results in the presence of noise. Moreover, it is very fast and can be used to extract basic structural information from an image.

**Figure 5:**
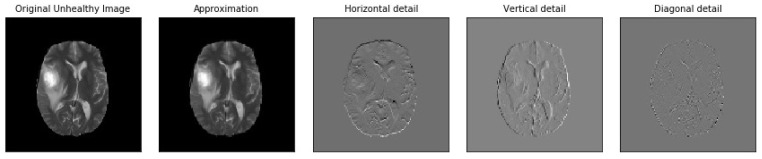
Samples of our Level 1 DWT decomposition of a non-healthy MR image type T2.

**Figure 6:**
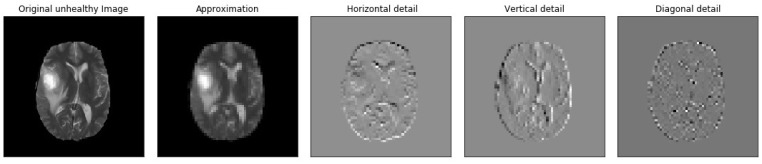
Samples of our Level 2 DWT decomposition of a non-healthy MR image type T2.

**Figure 7:**
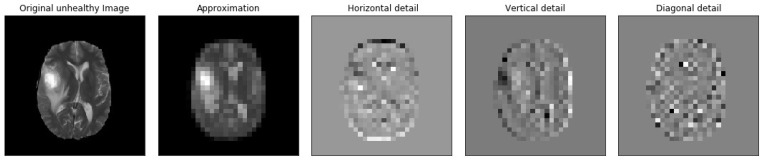
Samples of our Level 3 DWT decomposition of a non-healthy MR image type T2.

**Figure 8:**
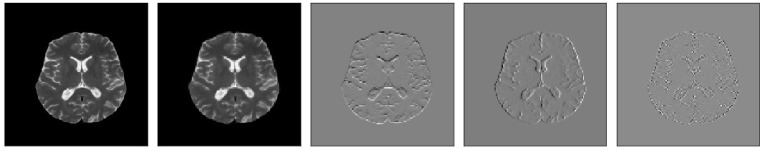
Samples of our Level 1 DWT decomposition of a healthy MR image type T2.

**Figure 9:**
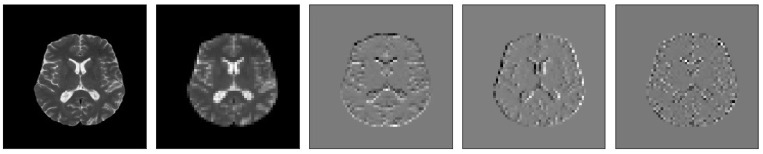
Samples of our Level 2 DWT decomposition of a healthy MR image type T2.

**Figure 10:**
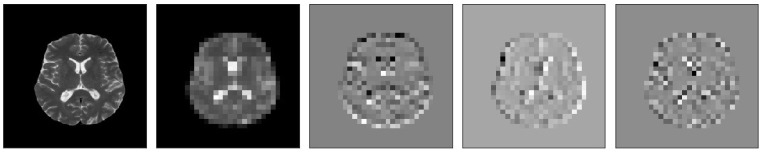
Samples of our Level 3 DWT decomposition of a healthy MR image type T2.

Figure 5, Figure 6, Figure 7, Figure 8, Figure 9 and Figure 10 visualize the sub-bands of each decomposition level by plotting each sub-band as a 2D image.

Our MR images analysis by DWT is calculated using Equations (10) and (11). We applied zero padding to coefficients of level 2 and 3 because we wanted equal size to all the coefficients (120,120).

#### 2.4.1. CNN2 Architecture

**Figure 11:**
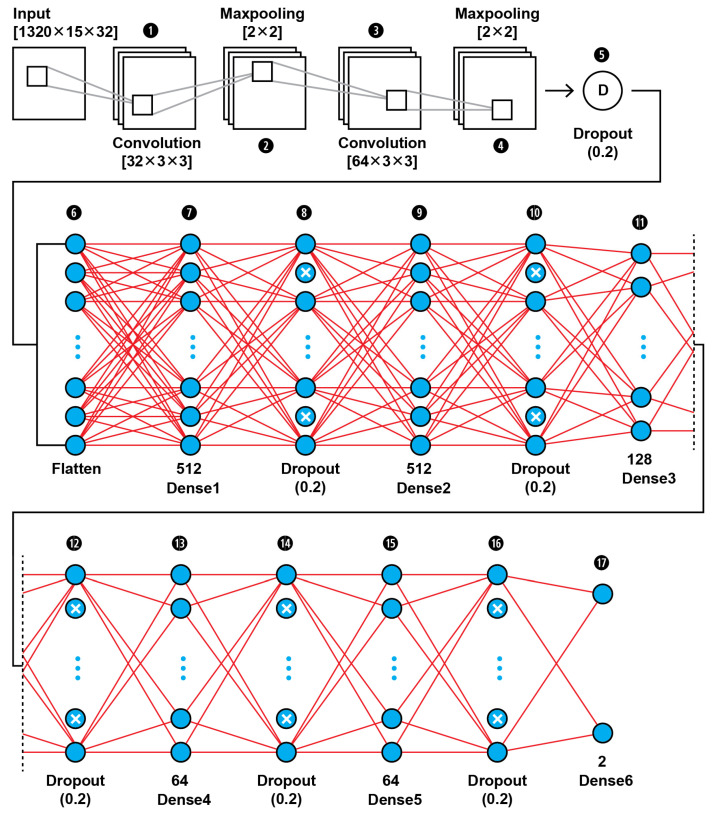
The proposed architecture of our 17-layer CNN2 using as input the computed DWT approximation coefficients of level 3 decomposition of MRIs, using as mother wavelet algorithm the simplest wavelet Haar.

The MRI features obtained by the 2D Haar 3 level DWT are fed into a CNN binary classifier. The topology of our CNN2 is shown in Figure 11 (as it is shown in the proposed approach in Figure 1); it was found to be the best fit for this classification task through trial and error. As shown in Figure 11, the input layer holds the augmented images as transformed to all the frequency components output from 3 level DWT in the dimensions of (1320,15,32).

In this 17-layer CNN architecture, there are 2 Convolutional layers with kernel size 3 × 3 and filter number 32 and 64. Each convolutional layer is followed by a MaxPooling layer of size 2 × 2 and before the classification phase begins there is a Dropout layer which is dropping out nodes with a possibility of 0.2. In the classification phase, there are 6 Dense layers of sizes 512, 128, 64 and 2. After each Dense layer a Dropout layer with 0.2 possibility of dropping a node follows. Dropout layers serve as allies in overfitting prevention.

Our CNN2 configuration is shown in Table 5. The hyperparameters are set before the training operation by us. The hyperparameter optimization used for this training is presented in Table 6.

#### 2.4.2. SVM Classifier Creation

Moreover, the MRI features obtained by the 2D Haar 3 level DWT are fed into a SVM binary classifier. The SVM is a supervised ML algorithm for binary classification by separating data points into two classes. An image classification problem can be solved by using the SVM. The objective of the SVM is to find a hyperplane that maximizes the separation of the data points to different classes in multidimensional space. We created our SVM by inputting our CNN2 conversion into an SVM by using the import l2 of tensorflow.keras.regularizers.

## 3. Evaluation Measures

### 3.1. Performance Metrics of a Single ML Model

To evaluate the performance of each of our binary classifiers, we compared the overall performance over the same data set, using accuracy, specificity, sensitivity, FPR, FNR, and precision metrics [55].

Accuracy (ACC) computes the probability of correctly recognizing a slice showing tumor as True Positive (TP) or True Negative (TN) among the total number of cases examined. It is calculated by Equation (8):

(8) $$\mathrm{Accuracy}=\frac{\mathrm{TP}+\mathrm{TN}}{\mathrm{TP}+\mathrm{FP}+\mathrm{TN}+\mathrm{FN}}$$

Sensitivity (SE) (also called the true positive rate, the recall, or probability of detection in some fields) measures the proportion of actual positives that are correctly identified as such (e.g., the percentage of tumor slices that are correctly identified as having the condition). It is calculated by Equation (9):

(9) $$\mathrm{Sensitivity}=\frac{\mathrm{TP}}{\mathrm{TP}+\mathrm{FN}}$$

Specificity (also called the true negative rate) measures the proportion of actual negatives that are correctly identified as such (e.g., the percentage of slices showing other than tumor diseases (or healthy slices) that are correctly identified as not showing tumor). It is calculated by Equation (10):

(10) $$\mathrm{Specificity}=\frac{\mathrm{TN}}{\mathrm{TN}+\mathrm{FP}}$$

False Negative Ratio (FNR), False Positive Ratio (FPR), and Precision are calculated by Equations (11) to (13):

(11) $$\mathrm{FNR}=\frac{\mathrm{FN}}{\mathrm{TP}+\mathrm{FN}}$$

(12) $$\mathrm{FPR}=\frac{\mathrm{FP}}{\mathrm{TN}+\mathrm{FP}}$$

(13) $$\mathrm{Precision}=\frac{\mathrm{TP}}{\mathrm{TP}+\mathrm{FP}}$$

where TP is the state when a slice showing tumor was correctly predicted as tumorous, TN is the state when a slice not showing tumor was correctly predicted as not tumorous, FP is the state when a slice not showing tumor was falsely predicted as tumorous, and FP is the state when a slice not showing tumor was falsely predicted as tumorous.

### 3.2. Comparing Performance of Two Binary ML Models

In the present study we compared the performances of pairs of models with the Edwards variant of the McNemar Test, the within-subjects chi-squared test, in order to examine if their variations are statistically significant or due to randomness. The chi-squared test compares the predictions of two binary (correct, incorrect) ML models to each other, paired through using the same test set. Instead of the list of false positive, true positive, false negative, and true negative counts of a single model, it is based on the layout of a the suiTable 2 × 2 confusion matrix (first row cells: A,B, second row cells: C,D). The C cell counts where model 1 is wrong and model 2 is correct. The B cell counts where model 2 is wrong and model 1 is correct. The discordant pair B,C, is a pair in which the outcomes differ for the pair A,D.

Our null hypothesis is that the two models do not differ, which means that cell B probability is equal to cell A probability. None of the two models performs better than the other. Thus, the alternative hypothesis is that the performances of the two models are not equal. Rejecting the null hypothesis, the performances of the two models are not equal.

We set the significance threshold to a = 0.05, and compared a’s *p*-value by assuming that the null hypothesis is true. The *p*-value is the probability of observing the given empirical x^2^ squared value. When the sample size in our scenario is relatively big (when *n* (=incorrect of model 1 + incorrect of model 2) is bigger than the recommended 25, *n* > 25), then x^2^ has a chi-squared distribution with 1 degree of freedom. However, in order to quantify if there is a significance between our models, we applied the corrected McNemar’s test as recommended by Edwards correction, [56] as follows:

(14) $$x^{2}=\frac{{(\left|“\mathrm{incorrect}”\;\mathrm{of}\;\mathrm{model}\;1-“\mathrm{incorect}\;\mathrm{of}\;\mathrm{model}”\;2\right|-1)}^{2}}{\left(“\mathrm{incorrect}”\;\mathrm{of}\;\mathrm{model}\;1+“\mathrm{incorect}”\;\mathrm{of}\;\mathrm{model}\;2\right)}$$

where x^2^ is the corrected McNemar statistic, “incorrect” are the off-diagonal cells B and C from the contingency table. B is the number of MR images that were detected correctly by model 2 and incorrectly by model 1. C is the number of MR images that were detected correctly by model 1 and incorrectly by model 2. For small sample sizes where B + C is smaller than the recommended 25, the chi-squared value may not be well-approximated by the chi-squared distribution. In case B > C, the two-sided *p*-value can be computed by Equation (15):

(15) $$p=2\sum_{i=b}^{n}\left(\begin{array}{c} n\\ i \end{array}\right)0.5^{i}{\left(1-0.5\right)}^{n-1}$$

where *n* = B + C, factor 2 is used to compute the two-sided *p*-value. The basic format for reporting a chi-squared test result is as follows:

X^2^ (degrees of freedom, N = sample size) = chi-squared statistic value, *p* = *p*-value.

Loss is defined as the cost of inaccurate predictions in the classification task. The Categorical Crossentropy Loss Function is employed for loss calculation. It computes the difference between target values and predicted values.

For the training procedure we used a balanced dataset of 764 scans originated by applying augmentation technique on the dataset of 190 non-tumorous cases and 190 selected patients with scans showing tumors.

We trained the models using ‘binary cross entropy’ as loss function and ‘RMSprop’ with learning rate 0.0001, as optimizer. The Loss function is calculated by Equation (16) [57]:

(16) $$H(t,p)=-\sum_{i=1}^{2}t_{il}\log(p_{i})=-[t\log(p)+(1-t)\log(1-p)]$$

where *t_i_* is the truth value taking a value of 0 or 1 and *p_i_* is the Softmax probability for the *i*^th^ class. The gradient of the weight associated with the loss function is obtained by the back propagation algorithm, and the weight is updated by the stochastic gradient descent.

The performance of the proposed CNNs is evaluated using fivefold cross-validation procedure for a Classification 1 task. The dataset is divided into five sets, out of which four sets are used for training and the remaining one is used for validating. The experiments are repeated five times. Classification performance for the task is evaluated for each set, and the average classification performance of the model is calculated.

The performances of our CNN1 and CNN2 are studied and compared: (i) case where CNN1 is fed by gray scale intensity pixel values, of each whole or from each quarter of each MRI, (ii) case where CNN2 is fed by the coefficients of two-dimensional 3 level DWT, of each MRI. Variations were tested using the original dataset or extended dataset by applying the augmentation technique. Moreover, the case is studied where PCA is applied or not on our input data. Here we mention only the best of our CNNs and they are compared with our SVM classifier. The tests of our models on unseen data are illustrated in Table 7.

**Figure 12:**
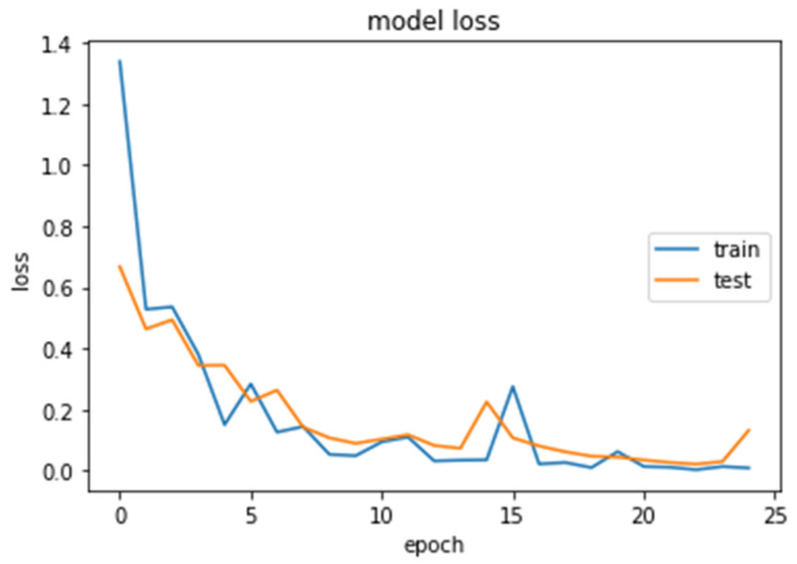
Loss (calculated by Equation (16)) of the CNN2–DWT model during training and testing of the CNN2 network.

**Figure 13:**
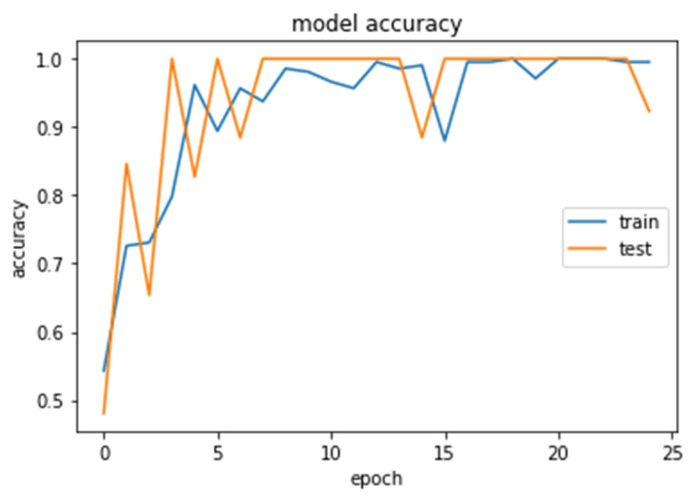
Accuracy (calculated by Equation (8)) of the CNN2–DWT model during training and testing of the CNN2 network.

**Figure 14:**
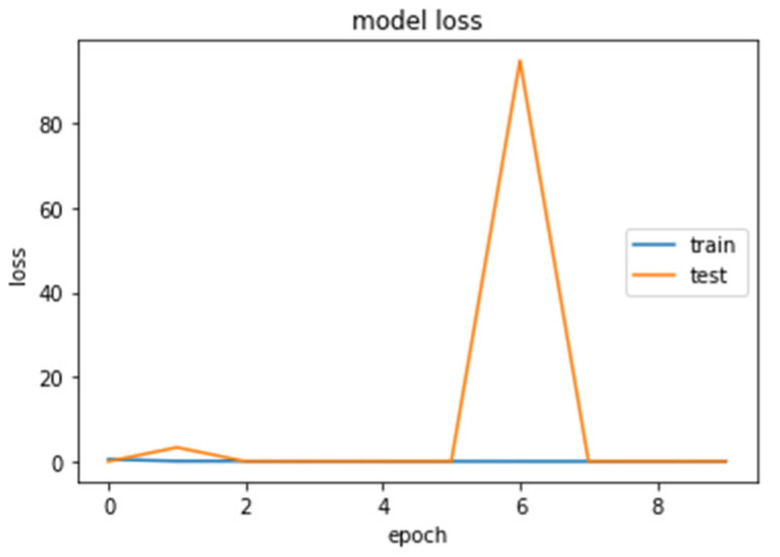
Loss (calculated by Equation (16)) of the CNN1 model during training and testing of the CNN1 network.

**Figure 15:**
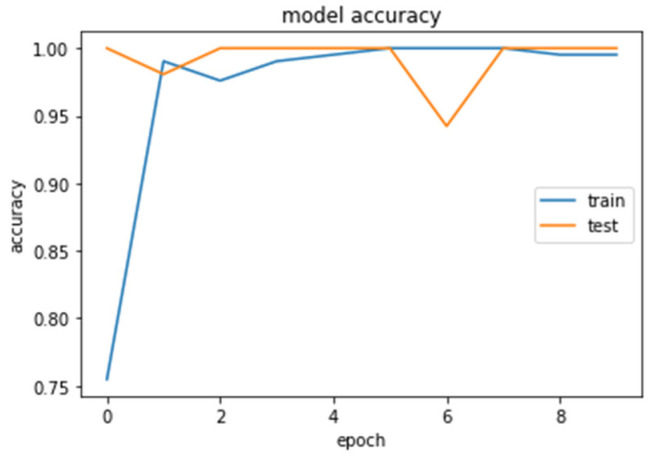
Accuracy (calculated by Equation (8)) of the CNN1 model during training and testing of the CNN1 network.

The accuracy and loss curves obtained while training and validating the network are demonstrated in Figure 12, Figure 13, Figure 14 and Figure 15.

## 4. Results

The CNN’s training and execution ran entirely in the cloud using Google Colaboratory, also known as Colab [58]. The Keras API Python library ran on top of the Tensorflow numerical platform in Python. The back propagation is automatically generated by TensorFlow.

### 4.1. CNN2 Down-Sampling Utilizing DWT

We analyzed our MRIs to DWT. Because we wanted equal size for all the coefficients (120,120), we applied zero padding to coefficients of levels 2 and 3 of DWT and next we standardized their values. The DWT coefficients as input data were fed to the CNN2 that was trained for 25 epochs, achieving an accuracy of 0.92. The model’s loss and accuracy while training are shown in Figure 12 and Figure 13.

### 4.2. CNN1 Down-Sampling Utilizing Average Pooling

We loaded our images (240,240) from the balanced dataset, next we normalized pixel values from 0–255 to 0–1. The pixel values as input data were fed to the CNN1. We trained the CNN1 model for 10 epochs, achieving an accuracy of 1.0. The model’s loss and accuracy while training are shown in Figure 14 and Figure 15.

### 4.3. SVM Classifier

We reshaped the pixel values matrix for each image, from (240,240) to a vector of 57,600 elements. After that, our dataset was in the form (57,600 pixels values in each row, Target). Next, we standardized our training set and we created a 10-fold split, in order to perform a grid search to find the optimal values of parameters ‘C’ and ‘kernel’ for the Support Vector Machine algorithm. We found out that optimal values were 0.7 for ‘C’ and ‘sigmoid’ for ‘kernel’. We applied the Support Vector Machine with those values to the training dataset, then we evaluated the algorithm on the test set, and we got an accuracy of 0.95.

### 4.4. Models Comparison

The results of the comparison using x^2^, chi-squared, test are presented in Table 8 and Table 9. In both cases, DF box (df = (NColumns − 1) ∗ (NRows − 1) = 1.

The chi-squared and *p*-value are computed as follows:

Calculating a chi-squared independence test on the Table 8 and Table 9, will verify if there is a significant difference in the performance of the two models.

The findings of the computed 2 × 2 contingency table of Model CNN–DWT and Model CNN, show that the x^2^, chi-square, from Equation (14), is equal to 149.7861, which yields the *p*-value *p* = 0.00001 and is smaller than the chosen significance level of α = 0.05. Thus, the null hypothesis is rejected. This means that there is a significant difference in the performance of the two models.

Similarly, the 2 × 2 contingency table of Model SVM-DWT and SVM shows that the x^2^, chi square, is equal to 73.5302, which yields the *p*-value *p* = 0.00001. This is smaller than the chosen significance level of α = 0.05; therefore, the null hypothesis is rejected.

Therefore, based on the accuracy and recall on comparison results, the CNN-DWT has significantly better performance compared to the CNN, and the SVM-DWT has significantly better performance compared to the SVM. CNN-DWT has significantly better performance compared to the SVM-DWT.

## 5. Discussion, Conclusions, and Future Work

Although T1 images are considered the foundation of MR imaging, due to the fact that they are those from which we derive structural information for pathology recognition, there is a kill switch in them; it is either there or not. You cannot pinpoint the type of pathology, despite the fact you know it is pathological, because the signal declines in the vast majority of cases (even though in rare cases it raises). Conversely, the T2 images show signal alterations due the presence or lack of fluids, and thus they can be used to better visualize edemas, tumor boundaries, presence of diaphragms, fusions, etc. All pathognomonic signs of tumors are related with the pathophysiology of the disease. In T2 imaging, MRI signal values have larger dispersion compared to the very narrow dispersion in T1, giving us a statistical advantage in the process of the training algorithm, due to the easier pattern recognition process. Moreover, T2 images are acquired in higher matrices enabling higher resolution, due to the signal creation process, which allows us to take advantage of the latest imaging techniques.

Direct comparison between T1- and T2-type MRI data, as input data to our CNN, could not be performed, because we did not have two series of images (T1, T2) belonging to the same family (FSE, SE, etc.) and holding constant imaging parameters for each series. The only way to be sure could be the creation of a tailor-made dataset.

The performance of the proposed DWT-CNN binary classifier of brain tumor T2 MRIs in two distinct classes, scans showing glioma tumors and scans showing other brain diseases or no diseases at all, is higher than the traditional CNN and SVM. Similarly, the SVM utilizing DWT instead of pixel intensities as inputs is higher.

Our DWT-CNN achieves 100% success in examining cases of non-tumorous people who are correctly identified as not having an ill condition, losing only 7% of the truly pathological slices showing illness. Our traditional CNN achieves 100% success in examining cases of sick people who are correctly identified as having an ill condition and 93% success in case of non-tumorous scans, who are correctly identified as not having this condition.

Although the CNNs and the DL models focus on automatic feature extraction from images, we claim that the use of functions for image transformation enhances the CNN training and can improve CNN performance in image processing. This claim is evaluated by comparing the performance of the CNN trained by images decomposed by DWT instead of raw images. As the images transformed by DWT functions are compressed, their capacity store is reduced. The negative point is that DWT of MRI decomposition increases the computational load.

The comparison of our results with other publications could be validated in the aspect of utilizing the same test data. The scans used for testing herein are mostly not distinct cases of tumors (as it is difficult to obtain information of positive malformations), for which our team’s radiologist felt that a lot time of careful observation and a lot of professional experience is required to obtain a diagnosis.

In case of MRIs with large and sharp tumor volumes, which were easy to recognize, we achieved 100% accuracy. Generally, the results obtained by the proposed CNN are characterized by very high performance values: our glioma classification accuracy is 97%, a little lower (−2.6%) than the best, 99.64% published by [22]. Recent results can be obtained from [22,59], where tabular references of recent relevant publications on the matter of classification of brain tumors based on CNNs are gathered.

In [60], Cinar et al. proposed a CNN from scratch, achieving accuracies of 99.64% for detecting glioma tumors, 96.53% for meningioma tumors, 98.39% for pituitary tumors, and 98.32% on average. Moreover, their results are better than those of deep CNN models based on transfer learning, such as ResNet50, VGG19, DensetNet121, and InceptionV3. In [61], Raaz et al. proposed a hybrid DL model for three types of brain tumors (glioma, meningioma, and pituitary tumor) by adopting a basic CNN architecture based on the GoogLeNet architecture. The proposed model obtained 99.67% accuracy, 99.6% precision, and 100% recall. In [62], Ozyurt et al. segmented the tumors using a Super Resolution (SR) Fuzzy-C-Means (FCM) approach for tumor detection from brain MR images, and then performed feature extraction and used pre-trained SqueezeNet architecture from smaller CNN architectures and a classification process with extreme ML. They obtained 98.33% accuracy, which is 10% higher than the rate of recognition of brain tumors segmented with FCM without SR. In [63], Deepak et al. classified three types of brain tumors using a pre-trained GoogLeNet to extract features from brain MR images with deep CNN and achieved 98% accuracy compared with the state-of-the-art methods. In [64], Cinar et al. proposed a hybrid ResNet50 CNN architecture by removing the last five layers of ResnNet50 and adding eight new layers; they obtained 97.2% accuracy, instead of the 92.53% accuracy obtained with the single ResNet50 model. In [65], Sajjad et al. proposed a CNN with the transfer learning method to detect brain tumor types by applying data augmentation and obtained 96.14%, 94.05%, and 93.21% accuracy for glioma, meningioma, and pituitary tumors, respectively. In [66], Ozyurt et al. proposed a detection method by segmenting the MRI tumor images using the NS-EMFSE method and by extracting features from the segmented images using AlexNet and then the ML model, KNN and an SVM, achieving 95.62% accuracy. In [67], Kaplan et al. extracted the nLBP and αLBP features and performed classification with KNN, random forest, ANN, A1DE, and linear discriminant analysis, achieving a highest accuracy of 95.56%. In [68], Swati et al. used the fine-tuned VGG-19 for the block-wise fine-tuning technique, achieving 94.84% classification accuracy in less training time than the hand-crafted features. Table 10 presents some of the latest relative studies in comparison to the present work.

Our next goal is to replace the end pooling layer by inverse DWT (IDWT) as an approach to recover the data details for image segmentation. Moreover, our plan is to add a next task, of another binary CNN classifier to discriminate the tumorous scans between low grade glioma and high grade glioma (under development by our team). Our future work focuses on the development of a set of ten binary CNNs in series, where each one will detect one of ten different diseases. The first CNN in the series will be yielding a very unbalanced binary problem, the 10th in the series will be yielding a balanced binary problem, and the intermediate ones are less unbalanced.

## Figures and Tables

**Table 1:** Presentation of the used data from 572 T2 MRIs separated into two subsets: Set1 with 382 scans and Set2 with 190 scans.

**Set1 (Training Set)**: Selected 382 T2 MRIs from 382 Patients ^3^*			
**Diagnosis**		**Patients**	**Augmented Scans (×2)**
Non-pathologica l ^1^*		168	2 × 168
Other disease (not tumor) ^1^*, ^2^*		151	2 × 151
Widened perivascular spaces	in 11 cases,		
Foci most probably associated with brain aging	12 cases,		
Hemorrhagic foci	9 cases,		
Ischemic changes and necrosis	32 cases,		
Hypoxic-ischemic changes	7 cases,		
Lesions associated with neurometabolic diseases	12 cases,		
Eclampsia	2 cases,		
Vasculitis	15 cases,		
Central pontine myelinolysis	9 cases,		
CNS degenerative diseases	12 cases,		
Multiple sclerosis	30 cases		
Tumor Glioma (Grade II, III, IV) ^2^*		34	2 × 34
Tumor Meningioma (Grade II, III, IV) ^2^*		20	2 × 20
Tumor Pituitary ^2^*		9	2 × 9
^2^* Set2 (Testing Set): Selected 190 T2 MRIs from 190 Patients			
**Diagnosis**		**Patients**	
Non-pathological ^1^*		10	
Other disease than tumor ^1^*^, 2^*		90	
Tumor Glioma (Grade II, III, IV) ^2^*		30	
Tumor Meningioma (Grade II, III, IV) ^2^*		30	
Tumor Pituitary ^2^*		30	

^1^* scans come from BraTS challenge, ^2^* scans come from Greek public St. George Hospital. ^3^* mean age: 34 years old (19–65).

**Table 2:** Hyperparameters setting of CNN1.

Hyperparameters	Setting
Loss function	ReLU, sigmoid
Optimizer function	Root Mean Square Propagation (RMSprop)
Metrics	binary_crossentropy
Epochs	30
Learning rate	0.0001
Dropout Rate	0.3
Number of Dense Nodes	2 to 1024

**Table 3:** CNN1 configuration.

Layer Name	Layer Type	Layer Configuration	Output Shape	Number of Parameters
Conv2D_1	Convolution	Number of Filters = 32—Kernel Size = (3 × 3)—Activation function = ReLU	(240,240,32)	320
Dropout_1	Dropout	Possibility = 0.3	(240,240,32)	0
Conv2D_2	Convolution	Number of Filters = 32—Kernel Size = (3 × 3)—Activation function = ReLU	(240,240,32)	9248
MaxPool2D_1	MaxPooling	Kernel size = (2 × 2)	(120,120,32)	0
Dropout_2	Dropout	Possibility = 0.3	(120,120,32)	0
Conv2D_3	Convolution	Number of Filters = 64—Kernel Size = (3 × 3)—Activation function = ReLU	(120,120,64)	18,496
Dropout_3	Dropout	Possibility = 0.3	(120,120,64)	0
Conv2D_4	Convolution	Number of Filters = 64—Kernel Size = (3 × 3)—Activation function = ReLU	(120,120,64)	36,928
MaxPool2D_2	MaxPooling	Kernel size = (2 × 2)	(60,60,64)	0
Dropout_4	Dropout	Possibility = 0.3	(60,60,64)	0
Conv2D_5	Convolution	Number of Filters = 128—Kernel Size = (3 × 3)—Activation function = ReLU	(60,60,128)	73,856
Dropout_5	Dropout	Possibility = 0.3	(60,60,128)	0
Conv2D_6	Convolution	Number of Filters = 128—Kernel Size = (3 × 3)—Activation function = ReLU	(60,60,128)	147,584
MaxPool2D_3	MaxPooling	Kernel size = (2 × 2)	(30,30,128)	0
Dropout_6	Dropout	Possibility = 0.3	(30,30,128)	0
Flatten	Flatten	-	115,200	0
Dropout_7	Dropout	Possibility = 0.3	115,200	0
Dense_1	Dense	Neurons = 1024—Activation function = ReLU	1024	117,965,824
Dropout_8		Possibility = 0.3	1024	0
Dense_2	Dense	Neurons = 512—Activation function = ReLU	512	524,800
Dropout_9		Possibility = 0.3	512	0
Dense_3	Dense	Neurons = 512—Activation function = ReLU	512	262,656
Dropout_10		Possibility = 0.3	512	0
Dense_4	Dense	Neurons = 128—Activation function = ReLU	128	65,664
Dropout_11		Possibility = 0.3	128	0
Dense_5	Dense	Neurons = 64—Activation function = ReLU	64	8256
Dropout_12		Possibility = 0.3	64	0
Dense_6	Dense	Neurons = 2—Activation function = sigmoid	2	130

**Table 4:** The basic two-dimensional DWT objects.

	Objects	Description
Image in original resolution	s A0 Ak, 1 ≤ k ≤ j Dk, 1 ≤ k ≤ j	Original image Approximation at level 0 Approximation at level k Details at level k
Coefficients in scale-related resolution	cAk, 1 ≤ k ≤ j cDk, 1 ≤ k ≤ j [cAj, cDj,..., cD1]	Approximation coefficients at level k Detail coefficients at level k Wavelet decomposition at level j

(where Dk stands for [Dk(h), Dk(v), Dk(d)] are the horizontal, vertical, and diagonal details at level k. The same holds for cDk which stands for [cDk(h), cDk(v), cDk(d)]).

**Table 5:** CNN2 configuration.

Layer Name	Layer Type	Layer Configuration	Output Shape	Number of Parameters
Conv2D_1	Convolution	Number of filters = 32—Kernel Size = (3 × 3)—Activation function = ReLU	(1320,15,32)	320
MaxPool2D_1	MaxPooling	Kernel size = (2 × 2)	(660,7,32)	0
Conv2D_2	Convolution	Number of filters = 64—Kernel Size = (3 × 3)—Activation function = ReLU	(658,5,64)	18,496
MaxPool2D_2	MaxPooling	Kernel size = (2 × 2)	(329,2,64)	0
Dropout_1	Dropout	Possibility = 0.2	(329,2,64)	0
Flatten	Flatten	-	42112	0
Dense_1	Dense	Neurons = 512—Activation function = ReLU	512	21,561,856
Dropout_2	Dropout	Possibility = 0.2	512	0
Dense_2	Dense	Neurons = 512—Activation function = ReLU	512	262,656
Dropout_3	Dropout	Possibility = 0.2	512	0
Dense_3	Dense	Neurons = 128—Activation function = ReLU	128	65,664
Dropout_4	Dropout	Possibility = 0.2	128	0
Dense_4	Dense	Neurons = 64—Activation function = ReLU	64	8256
Dropout_5	Dropout	Possibility = 0.2	64	0
Dense_5	Dense	Neurons = 64—Activation function = ReLU	64	4160
Dropout_6	Dropout	Possibility = 0.2	64	0
Dense_6	Dense	Neurons = 2—Activation function = sigmoid	64	130

**Table 6:** Hyperparameters setting of CNN2.

Hyperparameters	Setting
Loss function	ReLU, sigmoid
Optimizer function	Root-Mean-Square Propagation (RMSprop)
Metrics	binary_crossentropy
Epochs	80
Learning rate	0.0001
Dropout Rate	0.2
Number of Dense Nodes	2 to 512

**Table 7:** Models and classification reports.

Models Report						
Proposed Classifier	TP	TN	FP	FN	Total Correct	Total Wrong
Model 1: CNN-DWT	100	78	6	6	178	12
Model 2: CNN	95	84	0	11	179	11
Model 3: SVM-DWT	84	61	0	45	145	45
Model 4: SVM	67	106	17	0	173	17
Model 5: CNN-TL VGG16	74	92	17	7	166	24
**Classification Report**						
**Proposed Classifier**	**Accuracy**	**Sensitivity**	**Specificity**	**FPR**	**FNR**	**Precision**
Model 1: CNN-DWT	0.97	1.00	0.93	0.06	0.00	0.95
Model 2: CNN	0.97	0.94	1.00	0.00	0.05	1.00
Model 3: SVM-DWT	0.91	1.00	0.80	0.20	0.00	0.86
Model 4: SVM	0.79	0.63	1.00	0.00	0.36	1.00
Model 5: CNN-TL VGG16	0.87	0.91	0.84	0.14	0.08	0.86

**Table 8:** Contingency table for classifier comparisons on a sample of 190 MRI scans.

Contingency Table Model 4/Model 3	Model 3: SVM Correct TP	Model 3: SVM Wrong FN	Row Total
Model 4: SVM-DWT Correct TP	84	0	84
Model 4: SVM-DWT Wrong FN	45	67	112
Column total	129	67	196
X^2^ (1, N = 196) = 73.5302, *p*-value < 0.00001 The result is significant at *p* < 0.05			

**Table 9:** McNemar’s test for classifier comparisons on a sample of 190 MRI scans.

Contingency Table	Model 2: CNN Correct TP	Model 2: CNN Wrong FN	Row Total
Model 1: CNN–DWT Correct TP	100	6	106
Model 1: CNN–DWT Wrong FN	11	95	106
Column total	111	101	212
X^2^ (1, N = 212) = 149.7861, *p*-value = 0.00001 The result is significant at *p* < 0.05			

**Table 10:** Comparison of the proposed model with other recent studies.

Authors	Classification Type	Technique	MRI Type Data	Accuracy (%)
Cinar et al., 2022 [60]	CNN	Multi-class	T1-W	99.64
Raza et al., 2022 [61]	Deep CNN	Multi-class	T1-W	99.67
Ozyurt et al., 2020 [62]	SR-FCM-CNN	Multi-class	T1-W	98.33
Deepak et al., 2019 [63]	GoogLeNet	Multi-class	T1-W	98
Çinar et al., 2020 [64]	Hybrid ResNet50	Multi-class	T1-W	97.2
Sajjad et al., 2019 [65]	CNN–TL	Multi-class	T1-W	96.14
Ozyurt et al., 2019 [66]	NS-EMFSE AlexNet SVM KNN	Multi-class	T1-W	95.62
Kaplan et al., 2020 [67]	LBP SVM KNN	Multi-Class	T1-W	95.56
Swati et al., 2019 [68]	Deep CNN VGG-19	Multi-Class	T1-W	94.84
Present work	CNN-DWT	Binary (glioma)	T2	97

## Data Availability

The data used in this study are available on request from the corresponding authors.
